# The Impact of the COVID-19 Pandemic on Real-world Functioning in Adult Cochlear-implant Users

**DOI:** 10.1097/ONO.0000000000000048

**Published:** 2024-01-25

**Authors:** Tyler J. Young, Kara J. Schneider, Aaron Moberly, Terrin Tamati

**Affiliations:** 1 Department of Otolaryngology, Ohio State University Wexner Medical Center, Columbus, OH; 2 Department Otorhinolaryngology/Head and Neck Surgery, University of Groningen, University Medical Center Groningen, Groningen, the Netherlands.

**Keywords:** Cochlear implants, COVID-19, communication abilities, quality of life, social isolation

## Abstract

**Hypothesis:**

As a result of COVID-19 lockdowns and the associated effects on the auditory-social environments of cochlear-implant (CI) users, we expected that adult CI users would report a decrease in real-world communication abilities, a decrease in social isolation, and a decrease in quality of life (QOL) from pre- to post-pandemic.

**Background:**

The COVID-19 pandemic brought many changes to the environments in which adults with CIs interact and communicate. However, the impact of these changes on CI users’ real-world functioning is not well understood. This study investigated the impact of the COVID-19 pandemic on real-world communication abilities, social isolation, and CI-related QOL in adult CI users.

**Methods:**

Fourteen adult CI users completed self-report questionnaires assessing communication abilities, social isolation, and CI-related QOL at time points before and after the onset of the COVID-19 pandemic. Responses at the 2 time points were compared to evaluate changes in CI users’ real-world functioning.

**Results:**

Adult CI users showed a significant decrease in self-reported communication ability and a nonsignificant decline in CI-related QOL from before to during COVID-19. However, a nonsignificant trend of a decline in social isolation was also observed in adult CI users.

**Conclusion:**

Findings showed a decrease in self-reported communication abilities and, to a lesser extent, CI-related QOL, suggesting that changes to the auditory-social environment brought on by the COVID-19 pandemic may have negatively impacted communication abilities in real-world, challenging environments. Yet, the potential decrease in social isolation suggests that these changes may have had an overall positive effect on social interaction, potentially with close family and friends in well-controlled environments. Assessing changes in real-world functioning in the same CI users from both before and during the COVID-19 pandemic provided a unique glimpse into how changes in the auditory-social environment may impact outcomes in adult CI users.

Cochlear implants (CIs) provide a restored sense of hearing to adults with moderate-to-profound sensorineural hearing loss and ideally provide improved real-world communication abilities and social engagement. However, there are enormous individual differences in communication outcomes among adult CI users (1–5). Differences in pre-implantation hearing abilities, duration of hearing loss before surgery, and neurocognitive abilities, in part, explain some of the observed differences in communication outcomes (1,4). However, these individual factors are largely outside of patient or clinician control and may not be modifiable targets for impacting real-world functioning. The communication practices of adult CI users may represent a potentially modifiable factor that impacts communication outcomes. Vast individual differences in the real-world environments in which adult CI users communicate have been observed (6). Although not yet examined in adult CI users, previous research suggests that the types of everyday environments in which individuals experience speech and communicate (ie, the auditory-social environment), influence speech recognition abilities and broader real-world functioning. For example, social network size and diversity have been shown to influence speech processing in normal-hearing (NH) adults (7,8). In those studies, individuals with larger and more diverse social networks—providing substantial variability in speech input—show stronger speech recognition skills. Similarly, richer auditory-social environments may help CI users to develop and/or maintain stronger speech recognition skills. Further, beyond any perceptual benefit, opportunities to communicate with different people may also have a positive influence on an individual’s willingness and motivation to communicate in challenging environments and support greater overall well-being (9–12). Thus, any changes that impact the auditory-social environment in which CI users communicate may also influence their real-world functioning.

## COVID-19 and the Auditory-Social Environment

Studying changes to auditory-social environments and the subsequent impact on real-world functioning in adult CI users may provide insight into the role of the auditory-social environment in determining outcomes. The COVID-19 pandemic introduced numerous changes to the auditory-social environments of CI users, including population-level face masking, social distancing, and a decrease in the types of complex environments experienced on a daily basis (13,14). Using Ecological Momentary Assessment (EMA), Dunn and coworkers (2020) (13) found that CI users were immersed in quieter listening conditions during COVID-19 and were more likely to stay home or spend time outdoors. Similarly, Knickerbocker and coworkers (2021) (15) found that elderly patients who received CIs during the COVID-19 pandemic wore their CIs on average 1.2 hours less per day and spent less time listening to speech in noise compared with patients who had received their CIs before the COVID-19 pandemic.

These observed changes in auditory-social environments may have differing effects on speech recognition and broader real-world functioning. Interestingly, using retrospective questionnaires, the Dunn et al. (13) study found that the CI users reported less effortful speech recognition, as well as less social isolation and less anxiety due to hearing loss during the COVID-19 pandemic, compared with before the pandemic. However, some studies have found that CI users experienced increased difficulty and anxiety when trying to listen to others through face masks in public during the COVID-19 pandemic (14,16,17). Further, Knickerbocker et al (15) reported that CI users who had received their CIs during the COVID-19 pandemic did not achieve as much improvement in AzBio sentence recognition scores in the first 6 months following implantation compared with prepandemic CI users. Although this study examined the differences in AzBio scores in the context of missed/reduced programming visits, there may be other potential factors involved in determining outcomes. Taken together, these findings suggest that CI users may have been communicating in less complex and more controlled auditory-social environments during the COVID-19 pandemic, resulting in the perception of improved communication and social interaction at least within the controlled environments but potentially more difficulty in real-world, challenging settings.

## The Current Study

The goal of the current study was to further investigate how changes to the auditory-social environment brought on by the COVID-19 pandemic may have impacted CI users’ real-world functioning. This study examined self-reported real-world communication abilities, social isolation, and CI-related quality of life (QOL) both before and during the COVID-19 pandemic in the same CI users.

First, previous studies have suggested that at least some aspects of real-world communication in adult CI users may have been negatively impacted by the COVID-19 pandemic. In particular, the implementation of universal face masking in public has been shown to have caused considerable problems in communication in up to 80% of CI users (14,18). Given that the COVID-19 pandemic altered the nature of in-person communication, real-world communication abilities in adult CI users may have been even more broadly impacted. Thus, we expected that changes induced by COVID-19 on the auditory-social environment would lead to a decline in self-reported real-world communication abilities.

Second, a previous study indicated that social isolation levels among CI users may have decreased during the pandemic (13). One account of this finding is that CI users remained at home more often and communicated with family and friends in quieter environments as compared with in public spaces during the pandemic. In contrast, other studies suggest that NH individuals saw an increase in social isolation with the onset of the COVID-19 pandemic (19). In the current study, a measure of social isolation was included to provide converging evidence for the finding that CI users experienced a decrease in social isolation during the COVID-19 pandemic.

Finally, QOL among CI users has been shown to have declined from before COVID-19 to during COVID-19 (14). While some previous studies have focused on the impact of public masking on CI user’s reported QOL, here we used the Cochlear-Implant Quality of Life (CIQOL)-35 Profile instrument to evaluate overall CI-related QOL and individual domains of CI-related QOL (Communication, Emotional, Environment, Listening Effort, and Social) (20). Based on previous findings, we expected that changes induced by COVID-19 on the auditory-social environment would lead to poorer self-reported CI-related QOL.

## MATERIALS AND METHODS

### Participants

Fourteen CI users (6 male and 8 female), all of whom were postlingually deafened adults with at least 1 year of CI experience, participated in the current study. These individuals were originally part of a larger study on speech, language, and cognitive skills in adult postlingually deafened CI users and were invited to participate in the COVID questionnaires independently of their participation in the larger study. Demographic and hearing-related data for the CI users, obtained before the pandemic as part of a larger study, is provided in Table 1. At the time of the prepandemic testing session, the average age of the CI users was 66.78 (± 10.13) years. The average age at first CI was 55.62 (± 11.98) years, with an average duration of deafness before obtaining a CI of 26.38 (± 15.55) years, based on patient self-report of age when hearing loss began. To better understand the extent to which the participants were truly isolated to home versus still going to work and conversing with others during the pandemic, we include a breakdown of the occupations of the participants as follows: 9 participants were formally retired and 5 were employed, of which only 2 had professions that required continued interactions with others outside of the home during COVID. This study was approved by the Institutional Review Board (IRB) of The Ohio State University (IRB Protocol 2015H0173).

**TABLE 1.** Demographic and hearing-related information in adult CI users

Subject number	Sex	Age (years)	Age at implantation (years)	Duration of deafness (years)	Implant side	Implant brand	Contralateral hearing aid Usage	CID (% words correct)	Better ear PTA (dB HL)	Occupation
1	Female	69	54	24	Bilateral	Cochlear	Yes	90	120	Admin assistant
2	Female	58	44	31	Left	Cochlear	No	60	120	Retired
3	Female	73	56	54	Bilateral	Cochlear	Yes	84	112	Retired
4	Female	67	59	58	Right	Cochlear	No	70	115	Store manager
5	Male	63	57	4	Bilateral	Cochlear	No	92	120	Retired
6	Male	83	72	21	Bilateral	Cochlear	Yes	76	120	Retired
7	Female	70	58	31	Bilateral	Cochlear	Yes	90	120	Dog walker
8	Male	71	60	52	Left	Cochlear	Yes	92	80	Retired
9	Female	40	31	21	Left	Cochlear	No	76	116	Retired
10	Male	58	48	50	Bilateral	Cochlear	No	66	120	Retired
11	Female	66	43	60	Left	Advanced Bionics	Yes	46	missing	Retired
12	Female	70	Missing	Missing	Missing	Cochlear	No	80	120	Retired
13	Male	71	68	Unknown	Right	Cochlear	Yes	54	120	Kitchen supervisor
14	Male	76	73	33	Right	Cochlear	Yes	70	95	Retired

CID indicates Central Institute for the Deaf (CID) Auditory Test W-22 (Hirsh et al., 1952); PTA, unaided pure tone average across 0.5, 1, 2, and 4 kHz.

### Materials and Procedures

Self-report questionnaires of real-world functioning were administered both before and during the COVID-19 pandemic. Before the COVID-19 pandemic, paper versions of the questionnaires were completed between April 2019 and December 2019, as part of a larger in-person study. During the COVID-19 pandemic, online versions of the questionnaires were completed remotely between November 2020 and November 2021. While all 14 participants of this study had the opportunity to complete each of the 3 surveys exploring communication abilities, social isolation, and QOL, not all 14 participants completed each survey. Twelve completed the communication abilities and social isolation surveys and 10 completed the QOL survey.

#### Communication Abilities

Real-world communication abilities were assessed via the “Personal Assessment of Communication Abilities” (PACA) survey tool. The PACA survey is intended to be administered to gauge a participant’s perception of communication difficulties in common listening situations (21,22). Participants were presented with questions such as “How much difficulty do you have hearing in conversations of small groups?” and ranked their ability by selecting one of the following response options: “no difficulty” (1), “slight difficulty” (2), “moderate difficulty” (3), “quite a lot of difficulty” (4), “very much difficulty” (5), or “not relevant” (n/a). Total scores were calculated by taking the average score of all questions. Participants were asked to provide a response to all questions. However, In the case that the participant reported n/a or not relevant for one or more questions, average scores were calculated from the remaining questions. Higher scores represent greater self-reported real-world communication difficulty.

#### Social Isolation

The “Patient Reported Outcome Measurement Information System” (PROMIS Item Bank v2.0) social questionnaire was used to assess social isolation. The questionnaire evaluates the perceived quality of interpersonal relationships, social networks, companionship, feeling cared for, feeling valued, sense of belonging, and trust (23–25). Participants rate their current level of confidence in different social/community engagements by selecting one of the following response options: “very confident” (1), “quite confident” (2), “somewhat confident” (3), “little confidence” (4), and “not confident at all” (5). Participants were asked to provide a response to all questions. Overall scores were calculated by taking the average score overall questions. Higher scores represent greater self-reported social isolation.

#### Cochlear-Implant Quality of Life

CIQOL-35 Profile instrument was used to assess CI-related QOL (20,26,27). This questionnaire provides a global CIQOL score as well as 6 domains of CIQOL, including the communication, emotional, entertainment, environment, listening effort, and social domains. The CIQOL-35 profile is a well-validated and reliable tool to assess CI-related QOL. The profile utilizes 35 questions related to feelings of QOL with potential responses ranging from “never,” “rarely,” “sometimes,” “often,” and “always.” The global and domain scores were automatically generated using the CIQOL-35 automated scoring document, which produces a raw and converted score. Standardized scores (converted from raw scores) were used in the current study. Higher scores represent higher global and domain-specific QOL.

### Data Analysis

The scores of each outcome measure were subjected to a one-tailed, paired-comparison *t*-test with time point (before or during COVID-19) as the factor. One-tailed tests were used based on the prediction that the changes in the auditory-social environment brought on by COVID-19 would result in (1) poorer self-report communication abilities, (2) decreased self-report social isolation, and (3) poorer self-report CI-related QOL. For all measures, an alpha of 0.05 was set. To further explore the data, we also report the number of participants who demonstrate each trend (ie, increase/decrease in self-report functioning).

## RESULTS

### Communication Abilities

Self-reported communication abilities from before and during the COVID-19 pandemic were significantly different by the one-tailed, paired-comparison *t*-test (*t* [11] = 4.18, *P* = 0.001). Communication abilities were reported as less difficult (ie, lower scores) before the COVID-19 pandemic (M = 2.41, SD = 0.63) compared with during the COVID-19 pandemic (M = 2.78, SD = 0.52) by the adult CI users, suggesting an increase in communication difficulties during the COVID-19 pandemic. Note that these scores are still relatively low, indicating slight to moderate difficulty in real-world communication. Of the 12 adult CI users (6 male and 6 female) who completed the PACA questionnaire both before and during the COVID-19 pandemic, 9 (4 male and 5 female) reported an increase in communication difficulties, and three (2 male and 1 female) reported a decrease in communication difficulties. The communication abilities scores are shown in Table 2.

**TABLE 2.** Real-world functioning assessed before and during the COVID-19 pandemic in adult CI users

	Before COVID-19 (Mean ± SD)	During COVID-19 (Mean ± SD)	One-tailed, paired-comparison *t*-test
Communication abilities^a^ (n = 12)	2.41 ± 0.63	2.78 ± 0.52	*t*(11) = 4.18, *P* = 0.001
Social isolation^b^ (n = 12)	2.68 ± 1.03	1.99 ± 0.64	*t*(11) = 1.74, *P* = 0.23
CIQOL global^c^ (n = 10)	52.41 ± 6.63	50.68 ± 4.65	*t*(9) = 1.35, *P* = 0.011
Communication	50.25 ± 6.94	48.95 ± 7.35	
Emotional	68.10 ± 17.27	63.14 ± 14.10	
Entertainment	55.44 ± 15.73	53.10 ± 14.96	
Environment	66.74 ± 19.12	63.18 ± 13.86	
Listening effort	38.05 ± 10.77	39.10 ± 7.87	
Social	68.18 ± 16.95	63.39 ± 15.83	

There were 14 total participants in this study, however not every participant completed each questionnaire, hence the differences in subjects reported for each measure.

a Communication abilities, Higher scores represent more communication abilities difficulties.

b Social Isolation, Higher scores represent more social isolation.

c Cochlear-Implant Quality of Life (CIQOL), Higher scores represent higher CI-related QOL.

### Social Isolation

Self-reported social isolation was not statistically significantly different between the two timepoints by the one-tailed, paired-comparison *t*-test (*t* [11] = 0.77, *P* = 0.23). Although not significantly different, slightly greater social isolation was reported (i.e. higher scores) before (M = 2.10, SD = 0.66) compared to during (M = 2.00, SD = 0.64) the COVID-19 pandemic by the adult CI users. Note that these scores indicate relatively low social isolation among CI participants. Of the 14 total CI user participants, 12 adult CI users (6 male and 6 female) completed the PACA questionnaire both before and during the COVID-19 pandemic. Of these 12 participants (6 male and 6 female), 6 (5 male and 1 female) reported a decrease in social isolation, 5 (1 male and 4 female) reported an increase in social isolation, and 1 (1 female) showed no change. The social isolation scores are presented in Table 2 for the adult CI users.

### Cochlear-Implant Quality of Life

Standardized Global CIQOL scores (converted from the raw scores) at the 2 time points were not significantly different by the one-tailed, paired-comparison *t*-test (*t* [9] = 1.35, *P* = 0.11). However, slightly better Global QOL (ie, higher scores) was reported before (M = 52.41, SD = 6.63) compared to during (M = 50.68, SD = 4.65) the COVID-19 pandemic by the adult CI users. Of the 14 total participants of this study, 10 adult CI users (6 male and 4 female) completed the CIQOL questionnaire both before and during the COVID-19 pandemic. Of these 10 participants, 6 (3 male and 3 female) experienced a decrease in Global QOL, 2 (1 male and 1 female) experienced an increase in Global QOL, and 2 (2 male) experienced no change in Global QOL. The Global scores and scores on each subscale of the CIQOL are presented in Table 2 for the adult CI users.

## DISCUSSION

The current study examined the impact of changes in the auditory-social environment induced by the COVID-19 pandemic on real-world communication abilities, social isolation, and CI-related QOL in adult CI users. First, consistent with our prediction for communication abilities, we observed a significant increase in self-reported real-world communication difficulty from before to during the COVID-19 pandemic in adult CI users. Consistent with previous studies in which CI users reported increased listening difficulties with the onset of the COVID-19 pandemic, this finding suggests that the CI users experienced more difficulty communicating in real-world environments following the onset of the COVID-19 pandemic (14,18). The observed changes in self-report communication abilities may be explained in part by the impact of the COVID-19 pandemic on the environments in which CI users experience speech and communicate. One of the biggest changes was the introduction of nationwide face masking of the population. The attenuation of sound by the cloth and the inability to see auditory inputs such as lip and facial expressions may result in decreased speech recognition accuracy and increased effort (14). Further, an increased average distance away from the source of sound in public spaces due to social distancing may have made communication more difficult for CI users. These changes to the physical space in which CI users experience speech and communicate, among others, may have resulted in increased communication difficulty.

The social distancing rules introduced to mitigate the spread of the pandemic may have also led to a decrease in the quantity and quality of the speech experienced by CI users. Previous research suggests that the quantity and quality of speech input affect speech processing. More specifically, NH adults with larger social networks—providing more overall input and more diverse input—have been shown to display more accurate speech recognition compared to adults with smaller social networks, even when accounting for the amount of input (7,8). Experience with diverse input may lead to changes in the speech perception system that support speech recognition (7,8). Beyond this perceptual benefit, larger social networks may also have a positive influence on an individual’s willingness and motivation to communicate in challenging environments, further affecting real-world communication skills (14). In either case, limitations in the quantity and the quality of speech communication brought on by COVID-19 may have influenced CI users’ real-world hearing outcomes. Further, this finding could indicate that communicating and interacting with others in complex and challenging environments may benefit CI users’ communication abilities in real-world, challenging environments. Future research is needed to further evaluate how the auditory-social environment, communication practices, and potentially other malleable factors can impact communication outcomes in adult CI users.

Second, we observed a trend of decreased social isolation from before COVID-19 to during COVID-19 in adult CI users. Although the comparison did not reach significance, this trend is consistent with previous studies. Dunn et al (13) observed that 48 adult CI users reported less social isolation via questionnaires from pre-COVID to during the COVID-19 pandemic. In contrast to CI users, NH listeners were shown to have reported increased feelings of social isolation during the pandemic (28,29). One explanation for a potential improvement in social isolation among the CI users may be that more friends and family remained at home with the onset of the pandemic, providing more consistent social support and interaction within controlled listening environments. Thus, although CI users may have been experiencing overall less or altered communication outside the home (based on our results for communication abilities), they may have felt less isolated due to increased interaction with close friends or relatives within the home or other controlled environments. Further research exploring the factors affecting social isolation in adult CI users may help researchers and clinicians better explain and improve social engagement outcomes.

Finally, we observed a marginally small trend towards decreased CI-related QOL from before to during COVID. Although the comparison did not reach significance, 6 out of the 10 CI users who completed the questionnaire showed a decrease in CI-related QOL. This trend aligns with previous findings that showed a decrease in QOL among 221 adult CI users with the onset of the COVID-19 pandemic (14). Previous findings have also suggested NH adults have experienced a decline in QOL with the quarantine measures implemented during the COVID-19 pandemic (28). Several factors may have negatively impacted CI-related QOL, including the measures instituted to mitigate the spread of COVID-19 (ie, masking and social distancing) as well as its broader impact on social, psychological, and emotional well-being (30,31,32). Although these broader effects cannot be discounted, they are likely not solely responsible for the decrease in CI-related QOL. The CIQOL questionnaire used in the current study focuses on hearing- and communication-related aspects of QOL. Further, the “listening effort’‘ domain appeared to be most impacted by COVID-19. CI users showed an increase in listening effort with the onset of COVID-19, suggesting that the decrease in CI-related QOL may have been at least partly driven by communication-related challenges. Again, the trend in CI-related QOL in this study showed only a marginal decrease (52.41–50.68), and therefore conclusions drawn from this data need to be interpreted with this small decline taken into consideration.

Overall, although not significant, the observed trend of increased listening effort during the COVID-19 pandemic is not consistent with the findings of Dunn et al. (13) in which CI users reported exerting less effort during the pandemic than before it. In contrast with the Dunn et al. (13) study, the current study used questionnaires specifically targeting aspects of real-world functioning in the same group of participants before and during the COVID-19 pandemic (13). The repeat evaluation of questionnaires targeting specific real-world environments may be more sensitive to changes—or lack of changes—in individual CI users. Future research should more directly assess how changes to individual CI users’ auditory-social environment impact CI-related QOL, as well as how additional psychological, social, and emotional factors may further influence CI-related QOL.

### Limitations

Some weaknesses of this study should be noted. First, this study involved a small group of participants, which may have impacted the power of the study to find significant differences from pre- to during-COVID. Having a small group of participants may limit this study’s generalizability, but it also allowed for the testing of a unique and specific hypothesis that would otherwise be obscured by noise introduced by other factors in a larger participant group. Second, these participants frequently participated in our studies both before and during the COVID-19 pandemic, so they may have been more socially active than their peers. Additionally, participants completed the questionnaires online during the COVID-19 pandemic. As such, these participants had enough computer skills to suggest they may have had opportunities for communicating online that other CI users may not be able to access or navigate. Thus, the findings from the current study may not broadly generalize to other adult CI users. However, the differences (at least for communication abilities) observed in the current study may also be more exaggerated in a broader participant pool with less access to technology given that those CI users may not have had the opportunity to take advantage of electronic communication to connect with others. Third, these results observed in CI users may reasonably be expected to occur in hearing-impaired individuals without CIs. However, our group was most interested in exploring the impact of social-auditory environmental changes induced by COVID-19 on adult CI users because these individuals are at increased risk for communication difficulties and social withdrawal, due to the severity of their hearing loss before implantation as well as their reliance on the degraded signal delivered by the CI following implantation. Although CIs restore a sense of hearing to adults, listening remains challenging and effortful, which may lead to fatigue, social withdrawal, and poorer QOL. Finally, another potential limitation of this study is that the pandemic surveys were collected between the dates of November 2020 and November 2021. COVID-19 mask mandates and isolation restrictions varied by state and local jurisdiction in which participants resided. Therefore, the extent to which COVID-19 lockdowns may have impacted the participants may have differed depending on when the participants filled out the survey and where they resided. Despite these limitations, it is promising that the findings in this study were corroborated by previous studies using different measures and approaches. Further, compared to similar studies done before, a strength of this current study was that questionnaires were administered before and after the onset of the COVID-19 pandemic. Although the sample size is small, we were fortunate to be able to obtain these measures at these critical time points within the same group as part of a larger study. More research is needed from a larger study of more diverse hearing-impaired listeners with and without CIs to better understand how the auditory-social environment contributes to real-world communication outcomes.

## CONCLUSION

The goal of this study was to assess the impact of changes in the auditory-social environment induced by the COVID-19 pandemic on CI users’ real-world communication outcomes, including real-world communication abilities, social isolation, and CI-related QOL. Findings suggest that communication abilities were negatively impacted by the COVID-19 pandemic. Future research should continue to evaluate how changes in the auditory-social environment impact real-world communication outcomes in adults CI users. A better understanding of the impact that the auditory-social environment has on CI users would allow clinicians, researchers, and CI users alike to modify the environment and communication practices to improve real-world outcomes.

## ACKNOWLEDGMENTS

The authors would like to thank Jessie Lewis, Emily Clausing, and Ally Schmitzer for their assistance with this project.

## FUNDING SOURCES

Preparation of this manuscript was supported in part by funding from the President’s Postdoctoral Scholars Program (PPSP) at The Ohio State University, VENI Grant No. 275-89-035 from the Netherlands Organization for Scientific Research (NWO), and National Institutes of Health (NIH), National Institute on Deafness and Other Communication Disorders (NIDCD) R21DC019382 awarded to author T.N.T., as well as NIH NIDCD Career Development Award K23DC015539 and R01DC019088 to author A.C.M.

## CONFLICT OF INTEREST STATEMENT

Terrin N. Tamati and Aaron C. Moberly received grant support from Cochlear Americas for an unrelated investigator-initiated study. Aaron C. Moberly serves as a paid consultant for Cochlear Americas and Advanced Bionics, and serves as CMO for Otologic Technologies. No conflicts are declared by the other authors.

## DATA AVAILABILITY STATEMENT

The datasets generated during and/or analyzed during the current study are not publicly available, but are available from the corresponding author on reasonable request.
